# STATR: A simple analysis pipeline of Ribo-Seq in bacteria

**DOI:** 10.1007/s12275-020-9536-2

**Published:** 2020-01-28

**Authors:** Donghui Choe, Bernhard Palsson, Byung-Kwan Cho

**Affiliations:** 1 Department of Biological Sciences, Korea Advanced Institute of Science and Technology, Daejeon 34141, Republic of Korea; 2 Department of Bioengineering, University of California San Diego, La Jolla, CA 92093, USA; 3 Department of Pediatrics, University of California San Diego, La Jolla, CA 92093, USA; 4 KI for the BioCentury, Korea Advanced Institute of Science and Technology, Daejeon 34141, Republic of Korea; 5 Intelligent Synthetic Biology Center, Daejeon 34141, Republic of Korea

**Keywords:** Ribosome profiling, Ribo-Seq, NGS analysis

## Abstract

Gene expression changes in response to diverse environmental stimuli to regulate numerous cellular functions. Genes are expressed into their functional products with the help of messenger RNA (mRNA). Thus, measuring levels of mRNA in cells is important to understand cellular functions. With advances in next-generation sequencing (NGS), the abundance of cellular mRNA has been elucidated via transcriptome sequencing. However, several studies have found a discrepancy between mRNA abundance and protein levels induced by translational regulation, including different rates of ribosome entry and translational pausing. As such, the levels of mRNA are not necessarily a direct representation of the protein levels found in a cell. To determine a more precise way to measure protein expression in cells, the analysis of the levels of mRNA associated with ribosomes is being adopted. With an aid of NGS techniques, a single nucleotide resolution footprint of the ribosome was determined using a method known as Ribo-Seq or ribosome profiling. This method allows for the high-throughput measurement of translation *in vivo*, which was further analyzed to determine the protein synthesis rate, translational pausing, and cellular responses toward a variety of environmental changes. Here, we describe a simple analysis pipeline for Ribo-Seq in bacteria, so-called simple translatome analysis tool for Ribo-Seq (STATR). STATR can be used to carry out the primary processing of Ribo-Seq data, subsequently allowing for multiple levels of translatome study, from experimental validation to in-depth analyses. A command-by-command explanation is provided here to allow a broad spectrum of biologists to easily reproduce the analysis.

## OVERVIEW

Living organisms are sustained by incredibly complex biological processes, including DNA replication, metabolism, transcription, and translation. All biological processes are encoded in genomic DNA and transferred to proteins by messenger RNA (mRNA). Within these processes, the key element that defines the specific proteins that are to be expressed, and what amounts of proteins to produce in a certain situation, is transcription. To study the transcriptome, various transcriptome sequencing methods, such as RNA-Seq, TSS-Seq, and Term-Seq, have been developed (Nagalakshmi *et al.*, 2008; Sharma *et al.*, 2010; Dar *et al.*, 2016). Recent studies have reported that the transcript abundance is not directly correlated with protein levels in cells (Jeong *et al.*, 2016). Thus, a more direct method to measure the levels of cellular protein is required. An experimental method for isolating ribosome-protected mRNAs was devised in the early 1970s (Davies and Larkins, 1973). Ingolia *et al.* combined this method with NGS technology to determine the mRNAs that are being actively translated (Ingolia *et al.*, 2009). This method, called ribosome profiling or Ribo-Seq, allows for a relatively direct measure of gene expression compared to conventional transcriptome sequencing techniques. Furthermore, it provides a high-resolution map of the ribosome footprint, which allows for the determination of the protein synthesis rate, translational pausing, and translational response in cells in response to a variety of environmental changes. To investigate such a molecular-level understanding of translation, the data analysis step, as well as the experimental technique, is important. After the experiment and generation of data, the raw sequencing data is processed with a method similar to that used for the other sequencing data. Briefly, low quality reads, in-line control DNA, and sequencing adapter sequences are trimmed. The remaining high-quality reads are mapped on a reference genome sequence. A number of reads on each gene (or CDS) are counted and normalized to estimate expression levels. Ribo-Seq includes an additional step of detailed bioinformatic processing to decode the translational mechanism. However, the Ribo-Seq technique and analysis are used by a limited number of research groups and are not readily available for researchers who are new to the field. Here, we describe a simple analysis pipeline for Ribo-Seq in bacteria, denoted as simple translatome analysis tool for Ribo-Seq (STATR). STATR performs the primary processing of Ribo-Seq data, the results of which can then be used at multiple levels of translatome study, from experimental validation to in-depth analysis. STATR is open source, such that all the required files (example files, adapter sequences, and codes) are available at a public repository (https://github.com/robinald/STATR) under the GNU General Public License v.3.0 (https://www.gnu.org/licenses/gpl-3.0.en.html). In addition, the command-by-command explanation provided here can be used by a variety of biologists to easily reproduce our analysis.

## APPLICATIONS

Ribo-Seq provides a comprehensive understanding of translation in bacteria. It has been widely used to measure translation levels, as well as to investigate translational changes in response to diverse environmental stresses (Gerashchenko *et al.*, 2012; Shalgi *et al.*, 2013). Since Ribo-Seq accurately resolves the nucleotide-level profile of the ribosome, it provides a means to analyze ribosome pausing/stalling (Li *et al.*, 2012) and codon usage (Paulet *et al.*, 2017). In bacteria, it is believed that gene expression is regulated only transcriptionally since, unlike eukaryotes, bacteria lack complex post-transcriptional processes, including splicing, localization, and modification. In addition, translation and transcription are coupled in the cytosol. However, the presence and significance of translation have been highlighted recently, with regulatory processes being elucidated by Ribo-Seq (Van Assche *et al.*, 2015; Jeong *et al.*, 2016; Potts *et al.*, 2017). Novel proteins and upstream open reading frames (uORFs) have also been recently elucidated (Pelechano *et al.*, 2013). There are multiple widely-recognized tools for NGS analysis that are freely available with intensive documentation. These tools are memory-efficient, without the need for large computing power. Currently, it is possible to easily investigate high-throughput sequencing data in any modern computing environment. In addition, a virtual computing environment is an excellent alternative when physical space and the cost of equipment are not accessible. However, ones who are not familiar with bioinformatics and high-throughput sequencing data struggle with data analysis. The analysis pipeline described in this manuscript provides a simple and easy method for an initial analysis of Ribo-Seq and further in-depth studies of translation, if required.

## METHODS

### Ribo-Seq and recommended sequencing parameters

With respect to ribosomes remaining for different durations in different positions, the positional density of ribosomes varies within a transcript. This cellular variation can be analyzed experimentally using nuclease footprinting. First, to capture and isolate the ribosome-protected mRNA fragments, protein synthesis inhibitor antibiotics are used to efficiently freeze the ribosomes. Several methods can be used to isolate ribosome-protected mRNAs, including polyacrylamide gel electrophoresis (PAGE), followed by gel elution and size exclusion chromatography. Lastly, the isolated ribosome-protected mRNA fragments are subjected to sequencing library construction. Typically, the size of ribosome-protected mRNA fragments ranges between 26 and 34 nt. Thus, sequencing with a longer read cycle is not recommended. In addition, pair-ended sequencing is also unnecessary. Ribo-Seq is performed routinely using a single-ended 50 bp recipe on the Illumina platform, as in previous reports (Pelechano *et al.*, 2013; Jeong *et al.*, 2016; Choe *et al.*, 2019).

### Read trimming

**Figure 1.**
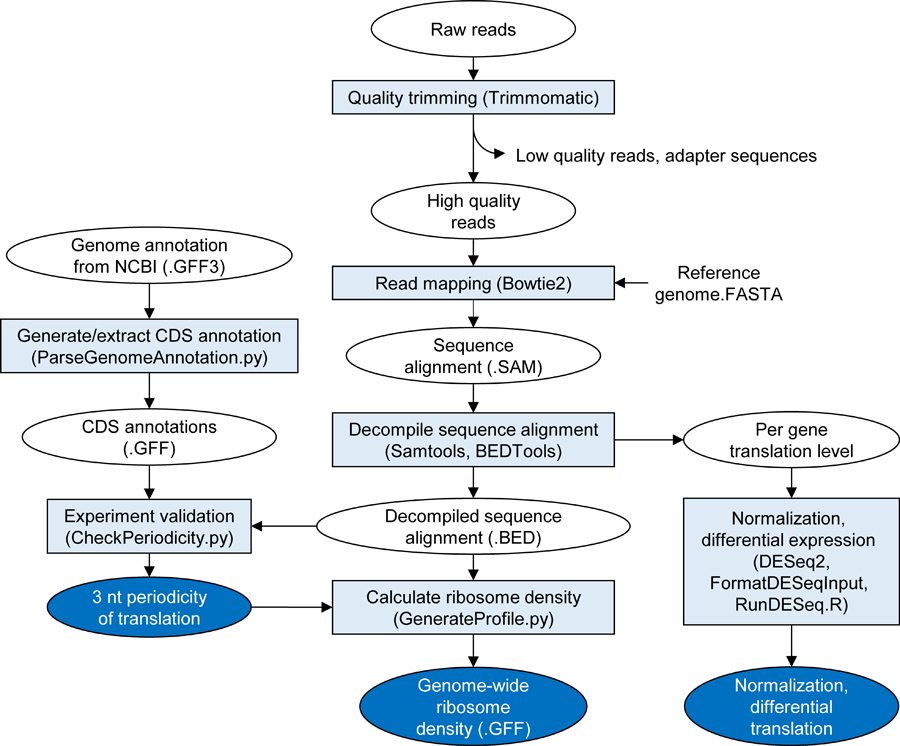
Overview of the STATR pipeline. Raw reads are quality trimmed, mapped, and decoded into interpretable data with series of software packages, python scripts, and R scripts.

The STATR pipeline comprises multiple steps of data processing (Figure 1). The analysis starts with the trimming of raw reads. The raw sequencing output file contains reads with quality scores for each base. The file contains reads with a variety of quality scores and may contain sequencing adapter sequences that are not removed during primary processing by the sequencer. The first step of the analysis involves removing the low-quality reads (or bases) and the adapter sequence that may not be removed from sequencer, so that only the high quality ribosome-protected sequence remains. Trimmomatic is a read processing tool designed for Illumina sequence data (Bolger *et al.*, 2014). Both the removal of low-quality reads and the adapter sequence can be carried out using Trimmomatic, generating trimmed reads ready for analysis.

### Mapping reads on reference

STATR uses Bowtie2 software to map trimmed reads to a reference genome sequence. Bowtie2 is a fast and sensitive read alignment tool. In Bowtie2, the long reference genome sequence is indexed into multiple partitions for memory-efficiency (Langmead and Salzberg, 2012). One output of Bowtie2 is the alignment information of all the reads in SAM file format. STATR includes the conversion step of SAM file into BAM file using Samtools (Li *et al.*, 2009), since the following pipeline (BEDTools) accepts only sorted binary format.

### Decompiling the sequence alignment

The mapping file is a compressed binary coded format of a SAM file containing all the information of quality and alignment for each of the reads to the reference. The file is a tab-delimited text that is readable, however, several codes (e.g. CIGAR string) are required to be further decompiled for downstream analysis. BEDTools is a robust tool for handling genomic file-formats including BAM and SAM files (Quinlan and Hall, 2010). In the STATR pipeline, two output files are generated by using BEDTools. One contains the number of reads aligned on each gene, which represents the translation level. Reads aligned only in the anti-sense strand of a gene are counted, since Ribo-Seq has strand-specificity. This translation level can be normalized and analyzed to determine the genome-wide translation level and differential translation in cells. The other output file contains the alignment information of individual reads throughout the reference genome. The result will be processed further to verify the integrity of Ribo-Seq and in-depth per base meta-analysis to gain biological insights.

### Experiment validation via meta-profile analysis

Ribo-seq provides a nucleotide-resolution footprint of the ribosome. Since the ribosome elongates the peptide chain discretely as decoding three-nucleotide code of mRNA, its footprint shows repeats of alternating dense-open peaks (Ingolia *et al.*, 2009; Mohammad *et al.*, 2019). Although alternative decoding events occur, such as frameshifting, hopping, and read-throughs (Chen *et al.*, 2014; Lang *et al.*, 2014; Fan *et al.*, 2017), the average ribosome density maintains a three-nucleotide periodicity. Thus, we exploited this characteristic to determine the quality of Ribo-Seq. However, Ribo-Seq in bacterial cells tends to shows less clear periodicity than that of eukaryotes (Mohammad *et al.*, 2019). In addition, the periodicity profile differs when the ribosome position is assigned to the 5’ or 3’ end of the reads, depending on the experimental conditions (Woolstenhulme *et al.*, 2015). The STATR pipeline calculates the average ribosome density over the CDSs to monitor the end that represents the ribosome position more precisely.

### Differential translation

In the previous processing step, the translation level (i.e. the number of mapped reads on every gene) was counted using BEDTools. However, this measure is a relative value, and needs to be normalized by sequencing depth in order to be compared sample-by-sample. DESeq2 is a model-based analysis tool that is widely used for transcriptome analysis (Love *et al.*, 2014). R scripts are included in this pipeline to examine the reproducibility of the experiment by accessing the hierarchical clustering of the samples and principal component analysis (PCA). After normalization and statistical analysis, data can be further analyzed to elucidate the specific responses of the experimental group with differentially translated genes.

## MATERIALS

### Equipment, software dependencies, and requirements

STATR runs on a Linux system. If a workstation or a computer cluster is not available, cloud computing is a cost-effective alternative. STATR requires a number of widely adapted tools, including Trimmomatic (v.0.39) for sequence quality control, Bowtie2 (v.2.2.6) for sequence alignment, Samtools (v.0.1.19) for convert/sort SAM file to binary coded BAM file, and BEDTools (v.2.25.0) for decompiling individual reads from the alignment (BAM file). Downstream scripts were intended to run on R (v.3.6.1) and Python (v.3.5.2). In addition, the following R packages are also required: DESeq2 (v.1.24.0), RColorBrewer (v.1.1–2), gplots (v.3.0.1), data.frame (v.1.12.6), optparse (v.1.6.4), and getopt (v.1.20.3).

## PROTOCOLS

### A. Conduct Ribo-Seq

The STATR pipeline analyzes Ribo-Seq data, wherein the example dataset used in this manuscript has been reported in previous studies (Latif *et al.*, 2015; Choe *et al.*, 2019). The dataset was generated following the standard experimental procedure. Briefly, 50 ml of culture was collected via centrifugation at 4,000 × *g*, 4°C for 10 min after 5 min of treatment with chloramphenicol (34 mg/ml) at an exponential growth phase. The pellet was resuspended with 0.5 ml of lysis buffer (1% Triton X-100, 34 μg/ml chloramphenicol, 133 mM of NaCl, 4.75 mM of MgCl_2_, and 19 mM of pH 7.5 Tris-HCl). The resuspension was flash-frozen with liquid nitrogen and lysed by pestle and mortar. Then, the cell lysate containing 10 μg of RNA was treated with 2000 gel units of Micrococcal Nuclease (MNase; NEB) at 37°C for 30 min. Polysomes were recovered from the MNase-digested sample by size-exclusion chromatography using Illustra MicroSpin S-400 HR Columns (GE Healthcare) followed by phenol:chloroform:isoamyl alcohol extraction. The ribosomal RNA was removed from 5 μg of polysome-protected RNA using a RiboZero rRNA Removal Kit (Illumina) according to the manufacturer’s instructions. rRNA-subtracted RNA samples were phosphorylated by treating 10U of T4 Polynucleotide Kinase (NEB) at 37°C for an hour and purifying with RNeasy MinElute columns (Qiagen). Sequencing libraries were prepared from the phosphorylated RNA samples using the NEBNext Small RNA Library Prep Set for Illumina (NEB) according to the manufacturer’s protocol. NGS was performed on an Illumina HiSeq 2500 instrument with a single-ended 50 cycles recipe.

### B. Prepare software and directory before analysis

**Figure 2.**
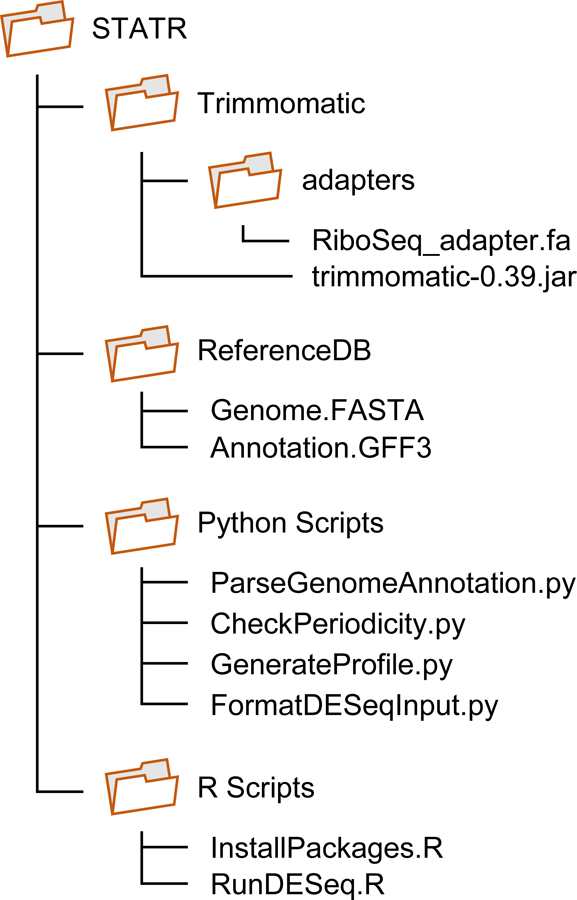
Default directory structure to run STATR. Default directory structure that supports running of STATR with a single Linux shell script. This structure is not mandatory.

The STATR command line is intended to be run on a directory structure, as shown in Figure 2. It can be modified freely by the user. However, the path should be modified accordingly.

1. Prepare Trimmomatic (v.0.39) by downloading and unzipping the binary file (available at http://www.usadellab.org/cms/index.php?page=trimmomatic). Trimmomatic runs on a Linux system with a working Java Development Kit (tested on v.1.8.0_222).
2. Install Bowtie2 (v.2.2.6) according to the instructions provided at http://bowtie-bio.sourceforge.net/bowtie2/index.shtml. One can install package to Linux as following.
  2.1. Download Bowtie2 source file (for example, bowtie2–2.2.6-source.zip) at http://bowtie-bio.sourceforge.net/bowtie2/index.shtml.
  2.2. Unzip the source file and use make command to build Bowtie2 as following.

User@Linux>unzip bowtie2–2.2.6-source.zip
User@Linux>cd bowtie2–2.2.6/
User@Linux>make
    ►By default, Bowtie2 builds with Threading Building Blocks (TBB) library. However, Bowtie2 can build without TBB if make fails. Use make NO_TBB=1 instead.
  2.3. Add executable files that have been made from **Step 2.2** to environment variable PATH. The simplest way of doing so is just copying all the executable files to the PATH as following.

User@Linux>sudo chmod +x <path to bowtie2>/bowtie2–2.2.6/
User@Linux>sudo cp /bowtie2–2.2.6/bowtie2 /usr/bin/
User@Linux>sudo cp /bowtie2–2.2.6/bowtie2-align-s /usr/bin/
User@Linux>sudo cp /bowtie2–2.2.6/bowtie2-align-l /usr/bin/
User@Linux>sudo cp /bowtie2–2.2.6/bowtie2-build /usr/bin/
User@Linux>sudo cp /bowtie2–2.2.6/bowtie2-build-s /usr/bin/
User@Linux>sudo cp /bowtie2–2.2.6/bowtie2-build-l /usr/bin/
User@Linux>sudo cp /bowtie2–2.2.6/bowtie2-inspect /usr/bin/
User@Linux>sudo cp /bowtie2–2.2.6/bowtie2-inspect-s /usr/bin/
User@Linux>sudo cp /bowtie2–2.2.6/bowtie2-inspect-l /usr/bin/
  2.4. Check installation by calling bowtie2. Make sure that printed version matches the version installed.

User@Linux>bowtie2 --version
3. Install Samtools (v.0.1.19) according to the instructions provided in http://www.htslib.org/ and **Step B.2**.
4. Install BEDTools (v.2.25.0) according to the instructions provided in https://bedtools.readthedocs.io/en/latest/ and **Step B.2**.
5. Install Python (v.3.5) according to the instructions provided in https://www.python.org/ and **Step B.2**.
6. Install R (v.3.6) according to the instructions provided in https://cran.r-project.org/doc/manuals/r-devel/R-admin.html/ and **Step B.2**.

### C. Trim low quality reads and adapter sequences

In this manuscript, we will demonstrate the pipeline using example dataset that is subsampled from previously reported Ribo-Seq result of *E. coli*, available through the Github repository (Choe *et al.*, 2019).

1. Locate the raw sequencing file (fastq.gz) in a directory named 1.Raw_data. Example files are available through the Github repository of STATR (https://github.com/robinald/STATR). Full files of previous studies are available via the European Nucleotide Archive (https://www.ebi.ac.uk/ena/data/view/PRJEB21199) (Choe *et al.*, 2019).
2. Locate the adapter sequence file (RiboSeq_adapter.fa) in Trimmomatic/adapters/directory. If using a different Ribo-Seq adapter than in previous studies (Latif *et al.*, 2015; Choe *et al.*, 2019), make a custom FASTA file containing the adapter sequence.
3. Trim the sequences using the following command:

User@Linux>mkdir -p ./2.Trimmed_reads
User@Linux>java -jar ./Trimmomatic/trimmomatic-0.39.jar SE -
phred33 ./1.Raw_reads/MG1655_RiboSeq_Sample1.fastq.gz ./2.Trimmed_
reads/MG1655_RiboSeq_Sample1_trimmed.fastq.gz 
ILLUMINACLIP:./Trimmomatic/adapters/RiboSeq_adapter_as.fa:2:30:6 
SLIDINGWINDOW:4:15 MINLEN:12 -threads 8
  ►SE: Single-end mode.
  ►-phred 33: Declare the quality scoring metric of the fastq file. Either Phred+33 or Phred+64. The recent pipeline of Illumina encodes the quality score with the Phred+33 format.
  ►ILLUMINACLIP: Although the sequencing adapter sequence is removed during the data generation step of the Illumina sequencing pipeline, the remaining adapter sequence will be removed once more. A considerable amount of raw reads contain an adapter sequence, because the size of the ribosome-protected mRNA fragment is very short (shorter than the sequencing cycle). Depending on the sequencing adapter used, custom sequences can be used if required. Three additional parameters follow in this option. The first parameter 2 is the maximum mismatches allowed in 16 nt seed sequence comparison. The second parameter 30 is for pair-ended reads and is omitted. The last parameter 6 is the minimum alignment score between the adapter and read for trimming. Ten matches are needed to score 6.
  ►SLIDINGWINDOW: Trimmomatic scans each read with a 4-bp sliding window. While scanning, reads are trimmed when the average quality of bases drops below 15.
  ►MINLEN: Very short reads can be non-specifically mapped to the genome. Theoretically, any 11-nt long sequence can occur in a 4^11^-bp (approximately 4.19 Mbp) genome. Thus, handling reads whose length is shorter than 12-nt is impractical.
  ►-threads 8: This step is optional. Use multithreading to reduce the running time.

### D. Align reads to the genome

1. Download and locate the reference genome sequence file (NC000913.3.fa) in the directory, ReferenceDB.
  1.1. Find the reference genome from the NCBI Nucleotide Database (https://www.ncbi.nlm.nih.gov/nuccore).
  1.2. Download the genome sequence from the menu ‘Send to-Complete Record-File-FASTA’.
2. Make a reference database using the following command:

User@Linux>mkdir -p ./ReferenceDB
User@Linux>bowtie2-build -f 
NC_000913.3.fasta ./ReferenceDB/MG1655_genome
  ►-f: Declare the input sequence for building the reference database is plain FASTA file.
3. Align the trimmed reads on the reference genome as follows:

User@Linux>mkdir -p ./3.Aligned
User@Linux>bowtie2 -q -
U ./2.Trimmed_reads/MG1655_RiboSeq_Sample1_trimmed.fastq.gz -
x ./ReferenceDB/MG1655_genome -
S ./3.Aligned/MG1655_RiboSeq_Sample1_mapping.sam --local
  ►-q: Declare the input file is FASTQ format.
  ►-U: Declare the input file is single-ended file and provide the file path to the input file.
  ►-x: Provide the file path to the reference sequence built at **Step D.2**.
  ►-S: Write the output file in SAM file format with the followed file path.
  ►--local: It is important to use the local alignment mode rather than end-to-end, since some additional bases can be attached at the end of the sequence. This option allows for read alignment with dangling ends.

### E. Decompile alignment file

1. Convert the .SAM alignment file into a .BAM file format as follows:

User@Linux>mkdir -p ./4.Decompiled/
User@Linux>samtools view -
bS ./3.Aligned/MG1655_RiboSeq_Sample1_mapping.sam -
o ./4.Decompiled/MG1655_RiboSeq_Sample1_mapping.bam
  ►view: This command is for analyzing the input alignment. If no additional option is given, return all the alignments in SAM format text.
  ►-bS: This option specifies the BAM output format from the SAM input file.
2. Sort the .BAM file for further analysis:

User@Linux>samtools 
sort ./4.Decompiled/MG1655_RiboSeq_Sample1_mapping.bam ./4.Decompi
led/MG1655_RiboSeq_Sample1_mapping.sorted
  ►sort: This command sorts alignments by the leftmost point of a read in ascending order of genomic coordinate for downstream analysis.
3. Decompile the sorted alignment file (.BAM) using the following command:

User@Linux>bedtools bamtobed -
i ./4.Decompiled/MG1655_RiboSeq_Sample1_mapping.sorted.bam 
> ./4.Decompiled/MG1655_RiboSeq_Sample1.bed
  ►-i: This parameter specifies the input sorted BAM file, which will be decompiled into a BED file.
  ► Note that the second > mark denotes the redirection operator of Linux and in the command line, and not that a new line is started.

### F. Validate experiment and visualize genome-wide ribosome profile

**Figure 3.**
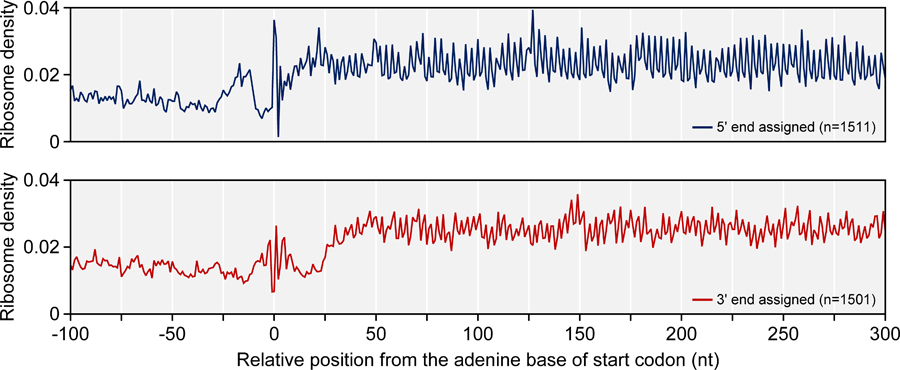
Average ribosome density on relative position from the start codon. Position of an adenine base of the start codon is 0. To assign ribosome position, 5’ or 3’ end of reads were examined. In this case, 5’ end position of reads shows clearer resolution than 3’ end assignment.

**Figure 4.**
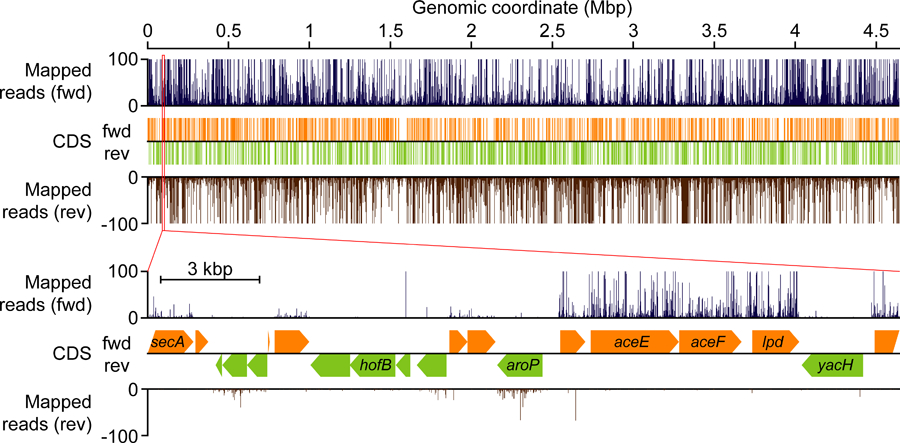
Genome-wide Ribo-Seq profile generated by the STATR pipeline and visualized by SignalMap (v2.0.0.5).

1. To validate the Ribo-Seq experiment, three-nucleotide codon periodicity will be tested. Genome annotation should be provided as a GFF file. STATR contains a python script that converts/flushes GFF3 annotation file downloaded from the NCBI database to a readable GFF3 file. The following steps should be performed:
  1.1. Find the reference genome from the NCBI Nucleotide Database (https://www.ncbi.nlm.nih.gov/nuccore).
  1.2. Download the genome annotation from the menu ‘Send to-Complete Record-File-GFF3’.
  1.3. Convert the information-rich GFF3 file into an essential GFF file as follows:

User@Linux>python ./Python_scripts/ParseGenomeAnnotation.py -
i ./ReferenceDB/NC_000913.3.gff3 -
o ./ReferenceDB/NC_000913.3_CDS.gff
    ►-i: This parameter specifies the input GFF3 file.
    ►-o: This parameter specifies the output file to generate.
    ►This python script extracts only CDSs from the input GFF3 and retrieves “locus_tag” as an identifier of CDS among many attributes in the input GFF3.
    ►Note that this step is optional if using custom genome annotations. Also, the GFF files can be freely customizable by the user in the text or in a spreadsheet editor software, such as Microsoft Excel.
2. Conduct meta-analysis using Python script, CheckPeriodicity.py, as follows:

User@Linux>mkdir -p ./5.MetaAnalysis/
User@Linux>python ./Python_scripts/CheckPeriodicity.py -
i ./4.Decompiled/MG1655_RiboSeq_Sample1.bed -
a ./ReferenceDB/NC_000913.3_CDS.gff -
o ./5.Meta_analysis/Meta_positional_density.txt
  ►-i: This parameter specifies the decompiled BED file from the previous section, **Step E.3**.
  ►-a: This parameter specifies the CDS annotation file in **Step F.1**.
  ►-o: This parameter specifies the output file to generate.
3. The output file is comprised of tab-separated information of meta-ribosome density. Check the three-nucleotide periodicity, as in Figure 3.
  ►Note that the position of adenine of the start codon is 0 rather +1, unlike general biological convention.
4. Generate a genome-wide Ribo-Seq profile using one of the two ends of the reads. The following python script generates a genome-wide Ribo-Seq profile with a user-selected end. The profile is a read count on each position of the genome:

User@Linux>python ./Python_scripts/GenerateProfile.py -
i ./4.Decompiled/MG1655_RiboSeq_Sample1.bed -e 5 -
a ./ReferenceDB/NC_000913.3_CDS.gff -n 1000000 -
o ./5.Meta_analysis/MG1655_RiboSeq_Sample1_Profile.gff
  ►-i: This parameter specifies the input decompiled BED file in **Step E.3**.
  ►-e: This option defines which end of the read is used for analysis. The user must select either the 5’ or 3’ end of the read to use, based on the resolution determined in **Step F.3**.
  ►-a: This parameter specifies the annotation file containing CDSs generated in **Step F.1.3**.
  ►-n: This parameter represents the normalization factor. If N is given, the script does not normalize the data. If a number is given, the script calculates how many reads may be mapped if there were a given number of reads mapped in total, similar to the TPM metric in RNA-Seq analysis (Li *et al.*, 2010).
  ►-o: This parameter specifies the output file to generate.
5. To visualize the profile multiple free software available, for example, SignalMap (https://sequencing.roche.com/en/products-solutions/by-category/target-enrichment/software/signal-map-software.html) and Integrative Genomic Viewer (http://software.broadinstitute.org/software/igv/) on the user’s own liability. By doing so, the user can freely inspect the genome-wide Ribo-Seq profile (Figure 4).

### G. Differential expression

1. To analyze the differential translation, count the mapped reads on every CDSs from the alignment file. Genome annotation should be provided as a GFF file. STATR contains a python script that converts/flushes the GFF3 annotation file downloaded from the NCBI database to a readable GFF file. First, download the genome information as follows:

User@Linux>mkdir -p ./6.Differential_expression
User@Linux>bedtools coverage -a ./ReferenceDB/NC_000913.3_CDS.gff 
-b ./4.Decompiled/MG1655_RiboSeq_Sample1.bed -s 
> ./6.Differential_expression/MG1655_RiboSeq_Sample1.cov
  ►-a: This parameter specifies the annotation file containing CDSs generated in **Step F.1.3**.
  ►-b: This parameter specifies the input decompiled BED file in **Step E.3**.
  ►-s: Ribo-Seq is a strand-specific RNA-Seq method. Sequenced reads are on the same strand as the gene. Thus, only reads with the same strand of CDS are counted by this option. Careful not to use -S (upper S) option.
  ►Note that the second > mark denotes the redirection operator of Linux and in the command line, and not that a new line is started.
2. Next, merge and re-format the read count into a single file that can be analyzed by the DESeq2 package. Use the python script, FormatDESeqInput.py, as follows:

User@Linux>python ./Python_scripts/FormatDESeqInput.py -
i ./6.Differential_expression/MG1655_RiboSeq_Ctrl1.cov:./6.Differe
ntial_expression/MG1655_RiboSeq_Ctrl.cov:./6.Differential_expressi
on/MG1655_RiboSeq_Exp1.cov:./6.Differential_expression/MG1655_Ribo
Seq_Exp2.cov -o ./6.Differential_expression/DESeq_Input.txt
  ►-i: This parameter specifies the coverage files calculated in **Step G.1**. Provide multiple files separated by a colon (:).
  ►-o: This parameter specifies the output file to generate.
3. Prepare an experiment design sheet. The experimental design sheet couples the given sequencing data files with each experimental condition, so that DESeq2 can identify which samples are replicates and which samples are required for comparison. The design sheet is a tab-separated text file with the following format:

File name: Design_Sheet.txt
File content:
Sample				Condition
MG1655_RiboSeq_Ctrl1		Control group
MG1655_RiboSeq_Ctrl2		Control group
MG1655_RiboSeq_Exp1		Experimental group
MG1655_RiboSeq_Exp2		Experimental group
4. Install the required R packages manually or by using InstallPackages.R as follows:

User@Linux>sudo Rscript ./R_scripts/InstallPackages.R
5. Run DESeq2 analysis with R script included in the STATR pipeline:

User@Linux>sudo Rscript ./R_scripts/RunDESeq.R -
i ./6.Differential_expression/DESeq_Input.txt -
d ./6.Differential_expression/Design_sheet.txt -
o ./6.Differential_expression/
6. The RunDESeq.R script generates multiple pdf files. First, Dendrogram_and_heatmap.pdf contains a dendrogram and a heatmap showing the hierarchical clustering of samples based on pairwise Euclidean distances between samples (Figure 5A). Also, the two-dimensional principal component analysis (PCA) plot is printed as PCA.pdf (Figure 5B). Based on the two graphs, the reproducibility of experiments can be examined. Then, a tab-separated text file comprising the DESeq2 normalized expression levels of each gene is printed as Normalized_expression.txt. Lastly, pairwise comparisons between given conditions are printed as Contrast_<Condition1>_vs_<Condition2>.txt files. The number of files is the same as the number of pairwise combinations of conditions (_N_C_2_; where N equals the number of experimental conditions including control). The comparison files contain the mean expression, log_2_ fold-change, and p-value statistics of the differential expression.

## EXPECTED RESULTS

**Figure 6.**
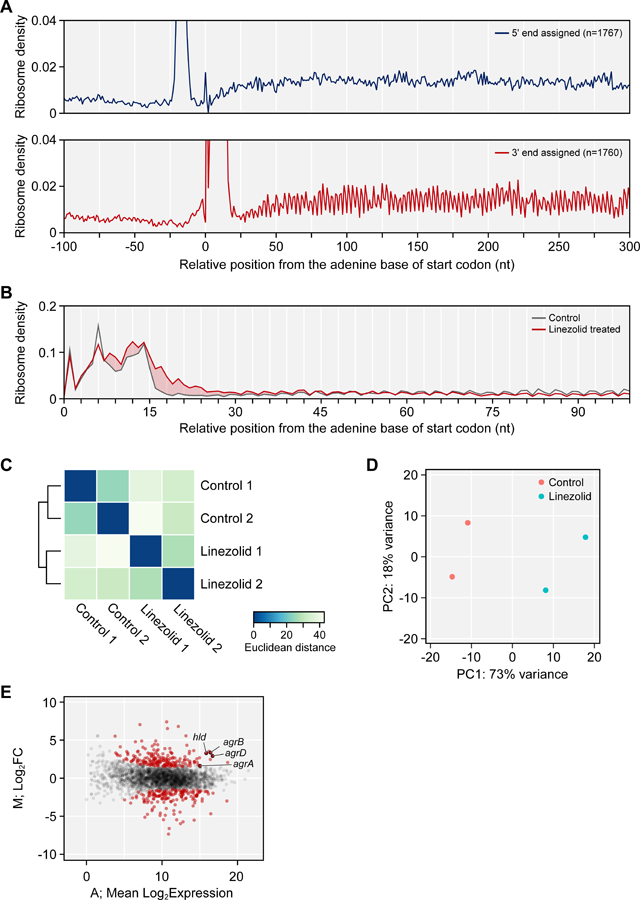
Translatome analysis of S. aureus Ribo-Seq using STATR. (A) Average ribosome density on relative position from the start codon. Ribosome position was inferred from 5’ or 3’ end of reads. Position of an adenine base of the start codon is 0. (B) Ribosome density of control and linezolid-treated S. aureus near start codon. Profile was colored red when RPF of linezolid-treated sample was higher than control sample. (C) The heatmap and dendrogram show pairwise distance between biological replicates. (D) Principal component analysis (PCA) of Ribo-Seq experiment. (E) MA-plot shows mean expression and expression change upon linezolid treatment. Each dots indicate individual genes. Differentially expressed genes (DEGs) are colored red.

Methicillin-resistance *Staphylococcus aureus* (MRSA) is multidrug resistant pathogenic bacteria that has become a major healthcare burden (Highlander *et al.*, 2007). To determine the virulence and resistance mechanism, we analyzed the translatomic response of the *S. aureus* USA300-HOU-MR strain under antibiotic treatment using the STATR pipeline. One of the oxazolidinone linezolid was treated with a sub-lethal dosage to reproduce the antibiotic treatment carried out in clinics. In the Ribo-Seq dataset, the 3’ end of the reads showed a clearer three-nucleotide periodicity than the 5’ end (Figure 6A). Interestingly, the ribosome density of the linezolid-treated *S. aureus* on close vicinity of the start codon was significantly higher than the control sample (Figure 6B). Linezolid is a translation initiation inhibitor that binds to the A site of the ribosome, preventing cognate-charged tRNA entering as a result (Renslo, 2010; Zhanel *et al.*, 2015). Considering that the A site of the ribosome lies ~14 nt upstream of the 3’ end of the RPF (Mohammad *et al.*, 2019), the increased RPF profile indicates ribosome stalling at the start codon (Figure 6B). As this example shows, Ribo-Seq analysis with STATR provides a nucleotide-resolution mechanistic understanding of translation *in vivo*. Next, we analyzed the differential translation of genes involved in the response toward linezolid treatment. Before analysis, the reproducibility between biological replicates and between the different treatment groups was examined. The two biological replicates were clustered well and the different experimental groups were separated distinctly (Figure 6C and 6D). By analyzing the normalized expression levels, we discovered 342 differentially expressed (translated) genes (DEGs) in which the expression level was significantly changed (adjusted *p*-value lower than 0.05) when treated with linezolid (Figure 6E). It has been reported that the transcription of the accessory gene regulator (Agr) is induced upon exposure to linezolid (Choe *et al.*, 2018). Agr proteins are the major regulators of the virulence and pathogenesis of *S. aureus* (Queck *et al.*, 2008), whose translation in response to linezolid has never been reported. DEG analysis revealed that the translational levels of Agr regulators are activated similar to the transcriptional levels (Figure 6E). Overall, STATR provides a simple and robust analysis pipeline for translational studies, including high-resolution ribosome footprinting, allowing for a molecule-level study of translation, as well as a genome-wide understanding of translation.

## Figures and Tables

**Figure 5.**
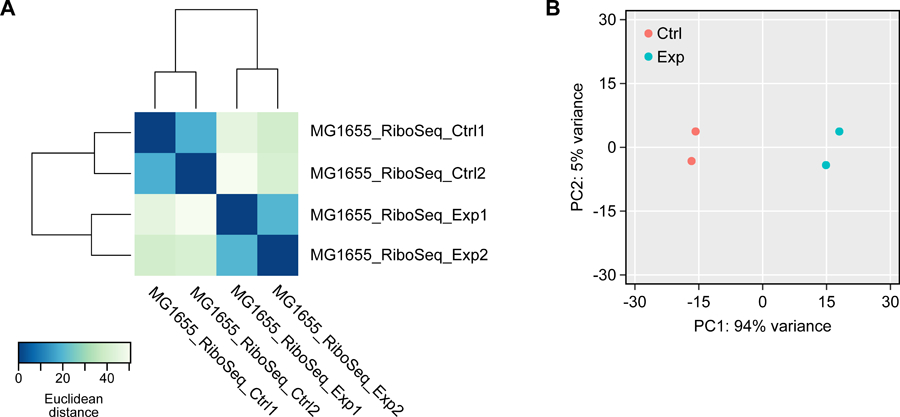
Heatmap and PCA plot generated by STATR for examining reproducibility of experiment. (A) The heatmap and dendrogram show pairwise distance between samples. (B) Principal component analysis (PCA) of Ribo-Seq experiment.
